# MRI and Ultrasound of the Thoracolumbar Fascia in the Setting of Degenerative Spinal Diseases

**DOI:** 10.3390/medicina62061045

**Published:** 2026-05-28

**Authors:** Noa Martonovich, Clara De Luca, Caterina Fede, Andrea Angelini, Pietro Ruggieri, Eyal Behrbalk, Carla Stecco, Carmelo Pirri

**Affiliations:** 1 Department of Orthopedic Surgery, Hillel Yaffe Medical Center, Hadera 3820302, Israel; noamartinowitch@gmail.com (N.M.); eyalb@hymc.gov.il (E.B.); 2 Rappaport Faculty of Medicine, Technion Israel Institute of Technology, Haifa 3200003, Israel; 3 Institute of Human Anatomy, Department of Neuroscience, University of Padova, 35122 Padova, Italy; clara.deluca@unipd.it (C.D.L.); carla.stecco@unipd.it (C.S.); carmelo.pirri@unipd.it (C.P.); 4 Department of Orthopedics and Orthopedic Oncology, University of Padova, 35128 Padova, Italy; andrea.angelini@unipd.it (A.A.); pietro.ruggieri@unipd.it (P.R.)

**Keywords:** low back pain, lumbar spinal stenosis, lumbar disc herniation, spondylolisthesis, ultrasound, magnetic resonance imaging, thoracolumbar fascia, thickness

## Abstract

*Background and Objectives*: The thoracolumbar fascia (TLF) has been implicated in low back pain, but imaging-based characterization in degenerative lumbar disorders, particularly in surgical cohorts, remains limited. To describe MRI-derived and US-derived TLF thickness estimates in a heterogeneous degenerative lumbar surgical cohort and explore preliminary imaging patterns and associations with selected clinical variables. *Materials and Methods*: In this prospective single-center cohort, adults scheduled for elective lumbar surgery underwent preoperative US (short- and long-axis at L3) and review of routine lumbar MRI (axial and sagittal T1-weighted measurements at L3) using standardized protocols. Twenty-six patients were included (15 with lumbar spinal stenosis, five with lumbar disc herniation, four with spondylolisthesis, and two with scoliosis). Disability was assessed using the Oswestry Disability Index (ODI). Exploratory subgroup comparisons, correlation analyses, and Bland–Altman agreement analysis were used to examine subgroup patterns, patient–factor associations, and MRI–US agreement. *Results*: Interpretable data were available for 19 axial MRI, 18 sagittal MRI, 19 short-axis US, 15 long-axis US, and 19 ODI assessments; paired MRI–US measurements were available in 11 cases for sagittal/long-axis analysis and 12 for axial/short-axis analysis. Mean TLF thickness was 0.89 ± 0.33 mm on axial MRI, 1.16 ± 0.48 mm on sagittal MRI, 2.53 ± 1.44 mm on short-axis US, and 2.49 ± 1.14 mm on long-axis US. Exploratory subgroup analyses showed a between-diagnosis difference only for axial-MRI-derived TLF thickness (*p* = 0.007), with lower thickness in disc herniation than in stenosis (*p* = 0.008), while sagittal MRI and US thickness measures did not differ between groups (*p* ≥ 0.301). ODI was not consistently associated with TLF thickness on MRI or US. *Conclusions*: In this exploratory surgical cohort, US-derived TLF thickness values were higher than those previously reported in the literature, suggesting possible fascial alteration in degenerative lumbar disease. However, TLF thickness was not consistently associated with disability, and MRI- and US-derived measurements should be interpreted as modality-specific estimates rather than interchangeable values. Given the small heterogeneous cohort and measurement constraints, these findings are descriptive and preliminary, but they provide an imaging framework to guide future standardized studies.

## 1. Introduction

Low back pain (LBP) is a major global burden. The number of people living with LBP increased by ~60% from 1990 to 2020, reaching an estimated 619 million people in 2020 and projected to rise to 843 million by 2050 [1,2]. While most cases are non-specific LBP (NSLBP), defined as pain not attributable to a specific pathoanatomical diagnosis, approximately 10–15% of cases are attributable to an identifiable spinal disorder, including, among others, stenosis, discopathies, and sagittal and coronal imbalance disorders [3,4].

Degenerative spinal disorders are traditionally framed through osseous and disc-related changes (e.g., facet arthropathy, intervertebral discopathies, degenerative coronal and sagittal imbalances [5,6,7]), yet symptoms and disability do not always map neatly onto structural findings [8,9,10]. This gap has increased interest in complementary pain generators and biomechanical contributors beyond the vertebra–disc complex.

The thoracolumbar fascia (TLF) is a multilayered aponeurotic complex investing the paraspinal musculature and mechanically linking the spine to the abdominal wall and pelvis [11,12]. Structurally, the TLF is formed by an anterior and a posterior layer: the anterior layer inserts medially onto the lumbar transverse processes, whereas the posterior layer attaches to the lumbar supraspinous ligament, envelopes the erector spinae as a paraspinal retinacular sheath, and fuses laterally into a thickened lateral raphe that blends with the abdominal aponeuroses [12,13]. At the microscopic level, these layers can be resolved into multiple collagenous sublayers with differing fiber orientations, separated by thin, loose connective tissue that permits interlaminar gliding [11].

Biomechanically, experimental loading demonstrates that tension applied via myofascial connections can deform the posterior TLF in a manner consistent with force transmission between the spine and lower limbs [14].

Immunohistochemical studies have demonstrated sensory innervation within the TLF, showing a dense network of free nerve endings, including nociceptive fiber populations with layer-specific distribution, supporting biological plausibility for the TLF as a pain-generating structure [15,16,17,18,19,20]. Experimental work further supports that nociceptive input from fascia can engage spinal nociceptive processing [21].

In vivo imaging studies in humans complement these findings: US elasticity imaging has shown reduced shear strain of the TLF in chronic NSLBP and increased TLF thickness compared with healthy controls [22,23,24]. However, substantial methodological heterogeneity has limited comparability across ultrasound studies. To address this, Pirri et al. introduced a standardized US protocol for TLF visualization and thickness assessment [24]. By contrast, MRI, despite being routinely used in patients with LBP, has been scarcely investigated for TLF assessment. Accordingly, the present exploratory study aimed to describe MRI-derived and US-derived TLF thickness estimates in a heterogeneous surgical cohort of patients with degenerative lumbar disorders, to illustrate preliminary imaging patterns and associations with selected clinical variables, and to provide a framework for future studies using standardized imaging approaches.

## 2. Materials and Methods

This prospective single-center study was conducted in accordance with institutional ethical standards. Formal Ethics Committee approval was not required based on the Italian Ministerial Decree of 30 November 2021. Written informed consent was obtained from all participants before enrollment and study-related assessments. Consecutive patients were recruited from the Orthopedic Department of the University of Padova.

Adults scheduled for elective lumbar surgery (decompression and/or fusion) between November 2024 and September 2025 were recruited and screened at preoperative admission the day before surgery.

Inclusion criteria: Age 18–80 years; male or female; chronic lumbar spinal pathology with clinical and radiological correlation, including lumbar spinal stenosis (LSS), lumbar disc herniation (LDH), scoliosis, and degenerative spondylolisthesis.

Exclusion criteria were known connective-tissue disorders or rheumatologic diseases affecting connective-tissue properties, active malignancy, and revision surgery.

Because MRI and US were acquired under different technical conditions and patient positionings, TLF thickness values derived from these modalities were considered modality-specific imaging estimates rather than directly interchangeable measures.

### 2.1. Clinical Assessment and Outcomes

At admission (preoperative), participants completed the Oswestry Disability Index (ODI), a validated 10-item instrument that quantifies low-back-pain-related disability on a 0–100% scale, with higher scores indicating greater limitation. ODI percentage scores were calculated by summing the scores of all completed items and multiplying the total by two [25].

### 2.2. Ultrasound Protocol

**Figure 1:**
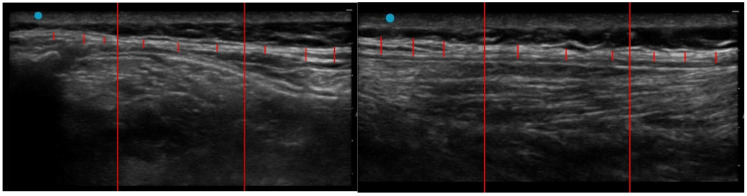
US assessment of TLF thickness at the L3 level. Representative short-axis (**left**) and long-axis (**right**) images obtained over the lumbar paraspinal region. Each image was divided into three equal mediolateral regions (vertical red lines). Within each region, TLF thickness was measured at three standardized points (red markers; 9 measurements per image) and averaged.

On the preoperative day, upon admission, participants underwent US assessment of the TLF. US was performed using a high-resolution system (Edge II, SonoSite, FUJIFILM, Bothell, WA, USA) with a 6–15 MHz linear transducer; the speed of sound was set to 1540 m/s (standard diagnostic setting) and B-mode depth to 30 mm. According to the Pirri et al. US protocol [24], the participants were positioned prone and relaxed, and the L3 level was localized by palpating the posterior superior iliac crest to identify L5 and then counting cranially by palpation of the spinous processes to L3. Images were acquired bilaterally at L3 with the transducer placed parallel to the spine, approximately 2–3 cm lateral to the L3 spinous process. For each side, one short-axis and one long-axis image were obtained. The entire acquisition protocol was then repeated by a second operator, yielding 8 images per participant (2 sides × 2 planes × 2 operators). From this image pool, a stratified random subset was selected for analysis (one short-axis and one long-axis image per side) and exported to ImageJ (available at https://imagej.net/ij/, accessed on 2 February 2026). TLF thickness was measured in ImageJ. To minimize local variability, each image was divided into three equal regions along the mediolateral axis. Within each region, three standardized measurement points (left, middle, right within that region) were used; thickness was measured perpendicular to the fascial plane at each point, and the three values were averaged to obtain a regional mean. The three regional means were then averaged to yield a single thickness value per image for analysis (Figure 1). Values measured in ImageJ were displayed to 3 decimal places (0.001 mm).

### 2.3. MRI Protocol

As part of the routine work-up, lumbar MRI (1.5 T) was obtained. T1-weighted sequences were analyzed using RadiAnt DICOM Viewer (64 bit, Medixant, Poznan, Poland), applying a standardized measurement protocol.

**Figure 2:**
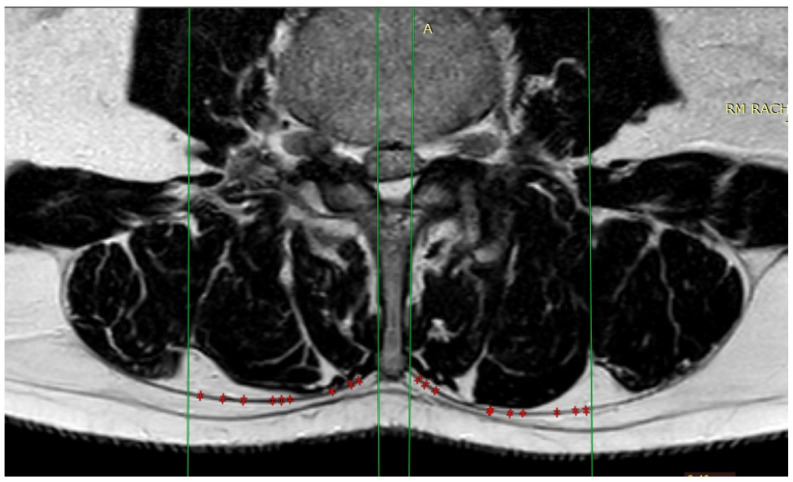
MRI assessment of TLF thickness in the axial plane at the L3 level. The green reference lines define the region of interest: one line drawn parallel to the spinous process (medial boundaries) and a second line at each side drawn at the longissimus–iliocostalis interface (lateral boundaries). The red markers indicate individual caliper measurements of TLF thickness with three measurements per third.

Axial plane: Measurements were performed at three L3 levels (superior endplate, mid-vertebral body, and inferior endplate). At each level, bilaterally, the region of interest was defined between (1) a line drawn parallel to the spinous process and (2) a line drawn at the longissimus–iliocostalis interface. This region was divided into three equal mediolateral thirds; within each third, thickness was measured at three predefined locations positioned at the inner thirds of that segment (left, middle, right) and averaged. The three regional means were then averaged to yield the value for analysis (Figure 2).

**Figure 3:**
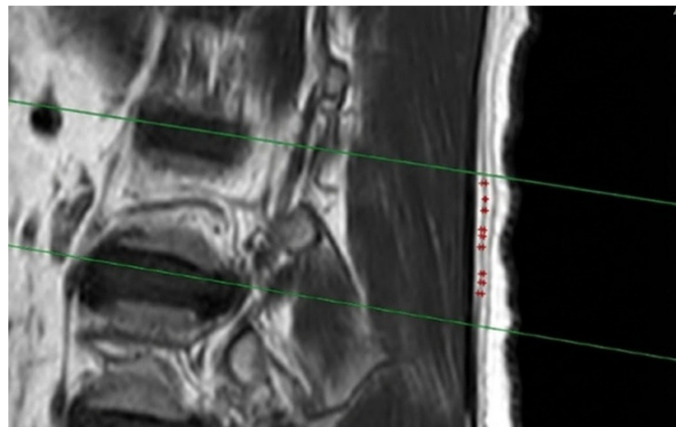
MRI assessment of TLF thickness in the sagittal plane at the L3 level. The upper and lower green lines mark the cranio-caudal boundaries of the L3 segment (above the superior endplate and below the inferior endplate). The red markers/short red lines indicate individual caliper measurements.

Sagittal plane: Two sagittal images immediately medial to the longissimus–iliocostalis border were selected. Two reference lines were drawn parallel to the superior and inferior endplates of L3, and the intervening region was divided into three equal thirds. Within each third, thickness was measured at three predefined locations (inner thirds: left, middle, right), averaged within each third, and then averaged across the three thirds to yield the value for analysis (Figure 3). Values measured in RadiAnt were displayed to 2 decimal places in millimeters (0.01 mm).

### 2.4. Statistical Analysis

Data were entered into Microsoft Excel and analyzed in JASP (v0.19.3). Continuous variables are presented as mean ± SD for approximately normally distributed data and as median (range) for non-normal data; categorical variables are reported as n (%). Normality was assessed using the Shapiro–Wilk test and Q–Q plots and homogeneity of variance with Levene’s test. Group comparisons were performed using Student’s *t* test or one-way ANOVA for approximately normal data (with Welch correction when variances were unequal), and Mann–Whitney U or Kruskal–Wallis tests for non-normal data, with appropriate post hoc testing and multiplicity adjustment. Correlations were assessed using Pearson’s r or Spearman’s ρ, according to distributional assumptions within each analytic subset, as the number of available observations differed across pairwise analyses. Two-tailed *p* values < 0.05 were considered statistically significant. Agreement between MRI- and US-derived TLF thickness was evaluated using Bland–Altman analysis, reporting the mean difference (MRI − US) and 95% limits of agreement; these analyses were restricted to participants with paired measurements available for the corresponding planes and were interpreted in the context of modality-specific measurement characteristics.

## 3. Results

### 3.1. Study Cohort

Twenty-six patients were eligible for inclusion. The cohort included 15 patients with LSS, five with LDH, four with spondylolisthesis, and two with scoliosis. Not all participants had analyzable data for every modality because imaging availability and interpretability differed across the preoperative work-up, and some studies did not include the required level or plane. Interpretable MRI measurements were available for 19 participants (19 axial and 18 sagittal), US measurements for 19 (19 short-axis and 15 long-axis), and ODI data for 19. Paired imaging was available in 11 patients for sagittal MRI and long-axis US and in 12 patients for axial MRI and short-axis US. The mean age was 54.23 ± 15 years (range 18–74), mean BMI was 26.75 ± 5.32 kg/m^2^ (16.41–39.04), and gender distribution was 58% male and 42% female. Ages were highest in stenosis (60.3 ± 10.0) and lowest in scoliosis (24.0 ± 8.5). Overall demographics are summarized in Table 1.

### 3.2. MRI-Derived TLF Thickness

In total, 19 lumbar MRIs were reviewed for LSS (n = 11), LDH (n = 5), spondylolisthesis (n = 2), and scoliosis (n = 1). Overall mean TLF thickness was 0.89 ± 0.33 mm (range 0.16–1.40) on axial images and 1.16 ± 0.48 mm (range 0.47–2.33) on sagittal images. Pathology-specific summaries are provided in Table 2.

**Figure 4:**
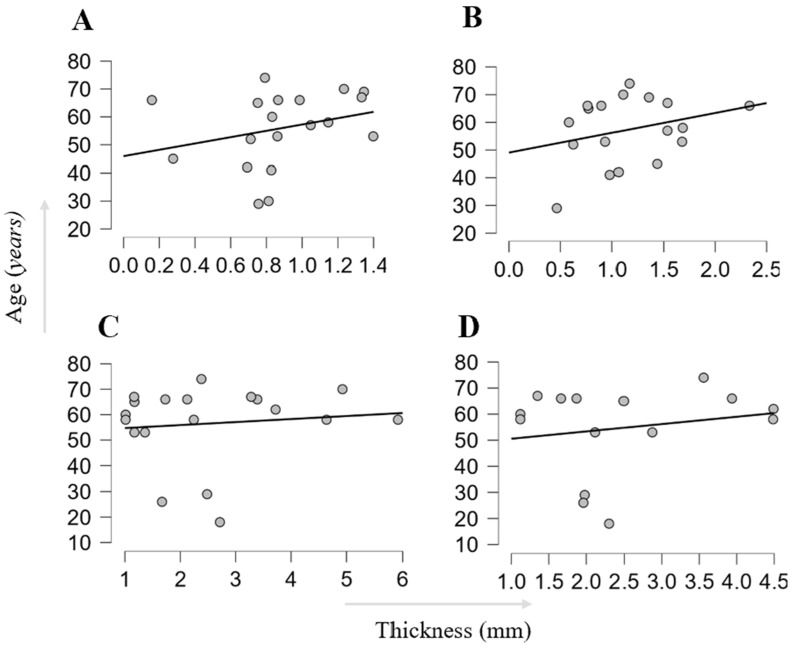
Scatterplots of age versus imaging-derived TLF thickness. Panels show (**A**) axial-MRI-derived TLF thickness, (**B**) sagittal-MRI-derived TLF thickness, (**C**) short-axis-US-derived TLF thickness, and (**D**) long-axis-US-derived TLF thickness. The *x*-axes represent TLF thickness (mm), and the *y*-axes represent age (years). Each dot represents one participant, and the solid line indicates the fitted linear trend. No statistically significant association was observed for axial MRI (Pearson’s r = 0.278, *p* = 0.249), sagittal MRI (Pearson’s r = 0.283, *p* = 0.255), short-axis US (Spearman’s ρ = 0.136, *p* = 0.580), or long-axis US (Pearson’s r = 0.191, *p* = 0.496).

**Figure 5:**
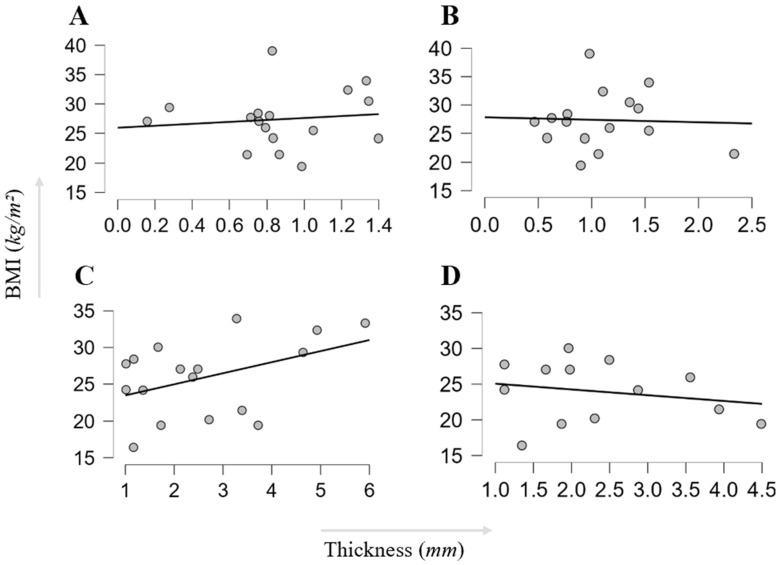
Scatterplots of BMI versus imaging-derived TLF thickness. Panels show (**A**) axial-MRI-derived TLF thickness, (**B**) sagittal-MRI-derived TLF thickness, (**C**) short-axis-US-derived TLF thickness, and (**D**) long-axis-US-derived TLF thickness. The *x*-axes represent TLF thickness (mm), and the *y*-axes represent BMI (kg/m^2^). Each dot represents one participant, and the solid line indicates the fitted linear trend. No statistically significant association was observed for axial MRI (Pearson’s r = 0.116, *p* = 0.658), sagittal MRI (Pearson’s r = −0.041, *p* = 0.881), short-axis US (Pearson’s r = 0.432, *p* = 0.083), or long-axis US (Pearson’s r = −0.206, *p* = 0.499).

**Figure 6:**
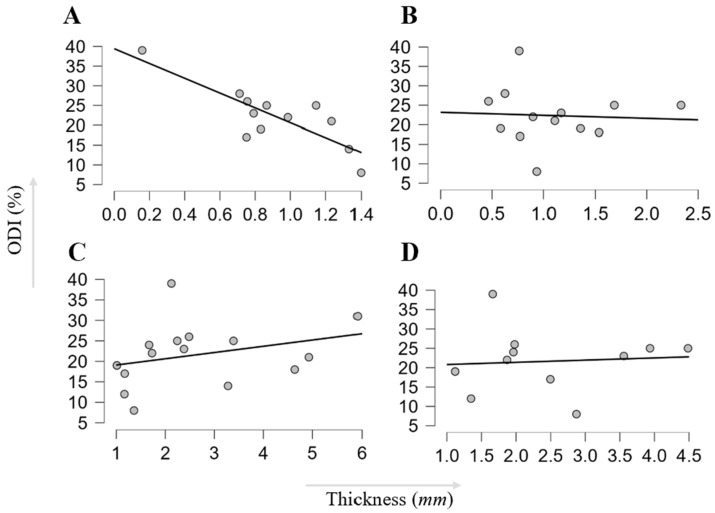
Scatterplots of ODI versus imaging-derived TLF thickness. Panels show (**A**) axial-MRI-derived TLF thickness, (**B**) sagittal-MRI-derived TLF thickness, (**C**) short-axis-US-derived TLF thickness, and (**D**) long-axis-US-derived TLF thickness. The *x*-axes represent TLF thickness (mm), and the *y*-axes represent ODI score (%). Each dot represents one participant, and the solid line indicates the fitted linear trend. ODI was significantly inversely correlated with axial-MRI-derived TLF thickness (Pearson’s r = −0.824, *p* < 0.001). No significant correlation was observed for sagittal MRI (Pearson’s r = −0.057, *p* = 0.853), short-axis US (Spearman’s ρ = 0.420, *p* = 0.105), or long-axis US (Pearson’s r = 0.082, *p* = 0.800).

No significant association was observed between age and MRI-derived TLF thickness on either plane (axial MRI: Pearson’s r = 0.278, *p* = 0.249; sagittal MRI: Pearson’s r = 0.283, *p* = 0.255; Figure 4). BMI was not significantly associated with MRI-derived TLF thickness on either plane (axial MRI: Pearson’s r = 0.116, *p* = 0.658; sagittal MRI: Pearson’s r = −0.041, *p* = 0.881; Figure 5). TLF thickness did not differ significantly by sex on either the axial (Welch’s *t*-test, *p* = 0.143) or sagittal MRI plane (Welch’s *t*-test, *p* = 0.366). ODI was inversely correlated with axial MRI TLF thickness (*p* < 0.001), whereas no significant correlation was found on the sagittal plane (Pearson’s r = −0.057, *p* = 0.853) as presented in Figure 6.

In exploratory subgroup analysis, axial-MRI-derived TLF thickness differed across diagnostic groups (one-way ANOVA, *p* = 0.007). Tukey HSD showed lower thickness in disc herniation compared with stenosis (*p* = 0.008) and spondylolisthesis (*p* = 0.039), while spondylolisthesis and stenosis did not differ (*p* = 0.873).

### 3.3. Ultrasound-Derived TLF Thickness

In total, 19 patients underwent US for LSS (n = 13), spondylolisthesis (n = 3), LDH (n = 2), and scoliosis (n = 1). Overall mean TLF thickness was 2.53 ± 1.44 mm (range 1.01–5.91) in the short axis and 2.49 ± 1.14 mm (range 1.12–4.49) in the long axis. Pathology-specific summaries are provided in Table 3. No significant association was observed between age and US-derived TLF thickness on either axis (short-axis US: Spearman’s ρ = 0.136, *p* = 0.580; long-axis US: Pearson’s r = 0.191, *p* = 0.496; Figure 4). BMI showed a non-significant positive association with short-axis-US TLF thickness (Pearson’s r = 0.432, *p* = 0.083), whereas no association was observed with long-axis-US thickness (Pearson’s r = −0.206, *p* = 0.499) (Figure 5). ODI was not significantly correlated with US-derived TLF thickness on either axis (short-axis US: Spearman’s ρ = 0.420, *p* = 0.105; long-axis US: Pearson’s r = 0.082, *p* = 0.800; Figure 6). On long-axis US, TLF thickness was significantly greater in females (mean 3.22 mm) than in males (mean 1.84 mm) (Student’s *t*-test, *p* = 0.013). Short-axis-US-derived TLF thickness did not differ significantly between diagnostic groups (one-way ANOVA, *p* = 0.301), and no significant between-group difference was observed for long-axis-US-derived TLF thickness (one-way ANOVA, *p* = 0.333), although descriptively stenosis tended to show higher values than the other groups.

### 3.4. Exploratory Comparison of MRI-Derived and US-Derived Measurements

Measurements derived from MRI and US showed substantial differences in mean values and ranges. Bland–Altman analysis showed that MRI produced lower TLF thickness values than US. For axial MRI versus short-axis US, the average difference was −1.191 mm, and for sagittal MRI versus long-axis US, the average difference was −1.789 mm (paired MRI–US samples are shown in Supplementary Tables S1 and S2).

## 4. Discussion

This exploratory study evaluated MRI-derived and US-derived TLF thickness as modality-specific imaging estimates in a heterogeneous surgical cohort with degenerative lumbar disorders and explored associations with patient factors and disability.

TLF thickness measured on MRI and US was not consistently associated with disability (ODI) in this cohort. An unexpected inverse correlation between ODI and axial MRI thickness (*p* < 0.001) was observed, but this finding was considered likely incidental. Prior US studies in chronic NSLBP have variably reported increased TLF thickness compared with asymptomatic controls [26,27]. This lack of a consistent association does not necessarily contradict the prior literature: in NSLBP, where no single structural diagnosis dominates, a subset of patients may have pain and disability more closely linked to myofascial factors, and a “fascial subgroup” has been proposed [28]. By contrast, in patients with defined degenerative spine pathology, ODI is likely influenced primarily by competing drivers such as neural compression, mechanical instability, and pain-chronicity-related factors. These dominant determinants can reduce the observable contribution of fascial morphology to functional limitations, making a correlation between TLF thickness and ODI less likely to emerge, particularly in small, heterogeneous surgical samples. While we observed an inverse correlation between ODI and axial-MRI-derived TLF thickness, the absence of a similar pattern in sagittal MRI and ultrasound suggests that this result may be incidental rather than reflective of a stable biological association. Accordingly, the present study cannot answer disease-specific or validation-level research questions but should instead be viewed as an exploratory study whose value lies in describing the imaging protocol, illustrating preliminary modality-specific patterns, and guiding future research.

When contextualized against the prior literature, in our degenerative surgical cohort, mean TLF thickness was 2.53 ± 1.44 mm in the short axis and 2.49 ± 1.14 mm in the long axis. These values are higher than those reported by Pirri et al., both in their NSLBP group (means ~1.95–2.13 mm short axis and ~2.09–2.27 mm long axis) and in healthy controls (~1.6–1.7 mm short axis and ~1.75–1.96 mm long axis) [24]. Taken together, this cross-study comparison raises the possibility that TLF thickness may be greater in some degenerative surgical cohorts than in patients with NSLBP, although differences in cohort composition and methodology preclude firm conclusions. On MRI, Caron et al. reported no differences in TLF morphology (e.g., layer lengths, circumference, and epimuscular fat contact) between individuals with chronic LBP and healthy controls and no association with pain severity [29]. In contrast, Adamietz et al. reported substantially greater TLF thickness than in our cohort—approximately twice our median axial values [30]. This divergence is likely driven, at least in part, by methodological differences in imaging and measurement definitions, such as MRI sequence, anatomical level and plane sampled, and whether only the posterior TLF lamina was measured or also the adjacent epimysium.

Wilke et al. [31] reported clear age- and BMI-related differences in US-derived TLF thickness. In our surgical degenerative cohort, associations with age were weaker overall and did not reach statistical significance. BMI in this cohort did not show an association with US- or MRI-derived thickness. These differences are plausibly explained by population and study-design differences.

While both US and MRI have been successfully used to assess deep fasciae (including the TLF), with good reproducibility reported for US thickness measures and demonstrated feasibility of MRI-based visualization [32,33], MRI-derived TLF thickness in our dataset is likely systematically underestimated relative to ultrasound due to acquisition constraints. All exams were performed at 1.5 T, where lower Signal-to-Noise Ratio (SNR) limits spatial resolution and blurs thin fascial interfaces, promoting conservative boundary tracing. MRI further relies on discrete slice sampling: maximal thickness may be missed if it falls between slices or if the plane is not orthogonal to the fascia. Partial volume effects are substantial, averaging TLF with adjacent muscle or fat and reducing apparent thickness. Additional inter-machine and protocol variability (slice thickness, reconstruction kernels, etc.) increases measurement dispersion and biases values downward [34,35]. By contrast, US provides continuous, real-time sampling and facilitates targeting of the maximal thickness but may overestimate due to inclusion of perimuscular connective tissue and angle-dependent anisotropy [36]. Overall, the observed MRI < US pattern is consistent with modality-specific bias, with MRI (particularly 1.5 T, non-standardized protocol) tending to underestimate TLF thickness.

In this cohort, ultrasound-derived TLF thickness values were higher than those previously reported in the literature, supporting the possibility of fascial alteration in degenerative lumbar disorders. Nevertheless, this finding was not consistently associated with disability, suggesting that increased TLF thickness should not be regarded as an automatic indicator of pain or functional impairment. By contrast, the main challenge appears to lie in MRI-based assessment, as routine lumbar MRI performed across different machines and with standard patient positioning is not specifically optimized for evaluation of such a thin fascial structure. Accordingly, MRI-derived thickness estimates should be interpreted with caution unless dedicated protocols and standardized positioning are applied.

Limitations: This study is limited by its single-center surgical design and the small, imbalanced diagnostic subgroups, which reduced statistical power for between-diagnosis comparisons and increased the risk of unstable estimates. Moreover, the cohort included heterogeneous spinal pathologies with differing severity, which may have confounded between-group comparisons. Consequently, the lack of between-group differences or associations between TLF thickness and other variables may reflect type II error, whereas isolated significant findings may represent type I error. Preoperative MRI and/or US were not available for all participants, for example, in patients who consented but could not tolerate the prone positioning required for ultrasonography or when the MRI did not include the study level, leaving imaging and clinical assessments restricted to cases with complete, interpretable data for each modality. Measurement validity is an additional limitation of this study. Because the thoracolumbar fascia is a thin structure, MRI-based thickness measurements may be influenced by spatial-resolution limits, slice orientation, partial-volume effects, and variability in clinical acquisition parameters [37]. Ultrasound measurements are also inherently operator- and technique-dependent, particularly with respect to probe pressure and angle [38]. Therefore, these measurements should be interpreted as standardized, modality-specific imaging estimates rather than as exact anatomical thickness values. Furthermore, all measurements were obtained by a single unblinded rater, and no formal inter-rater reliability analysis was performed, which may have introduced measurement bias. Additionally, the reported decimal precision reflected software display resolution rather than true measurement accuracy. These factors may have increased measurement variability and attenuated true associations. A further limitation is that the nature of the present study does not allow the research question to be answered definitively. The small, imbalanced, and biologically heterogeneous cohort precludes reliable disease-specific inference and does not permit formal validation of MRI against ultrasound. Its value is therefore exploratory: to describe the imaging approach, illustrate preliminary modality-specific patterns, and provide a framework for future studies in larger and more-homogeneous populations. Finally, the absence of a healthy control group limits inference about how thickness values compare with those in asymptomatic populations. Future studies should prioritize larger cohorts, standardized MRI sequences and US acquisition, and reliability testing and should expand imaging beyond thickness alone (e.g., echogenicity/texture measures on US, elastography, MRI-based characterization of adjacent tissue planes and signal features). Longitudinal follow-up could clarify whether baseline fascial imaging features relate to postoperative trajectories of pain, function, or treatment response. Future studies should evaluate whether a TLF-involved phenotype exists within degenerative lumbar disease and whether stratifying patients by fascial involvement improves clinical interpretability beyond diagnostic categories.

## 5. Conclusions

In this exploratory surgical cohort, ultrasound-derived TLF thickness values were higher than those previously reported in NSLBP and asymptomatic cohorts, suggesting possible fascial alteration in degenerative lumbar disorders. However, TLF thickness was not consistently associated with disability, and MRI- and US-derived thickness estimates should be interpreted as modality-specific measurements rather than interchangeable values. Given the small heterogeneous cohort and measurement constraints, the present study cannot answer disease-specific or validation-level research questions, but it provides a preliminary protocol and imaging framework to guide future studies in larger and more-homogeneous populations.

## Figures and Tables

**Table 1:** Demographic data by pathology.

Group by Pathology	N	Age		Gender	BMI		* ODI	
		Mean ± SD	Range	Male (%)	Mean ± SD	Range	Mean ± SD	Range
**Spinal stenosis**	15	60.3 ± 10.0	29–70	53	26.2 ± 5.7	16.4–33.9	41.0 ± 10.4	24–62
**Disc herniation**	5	55.8 ± 16.7	42–74	80	26.3 ± 2.9	21.4–29.3	60.0 ± 16.3	46–78
**Spondylolisthesis**	4	44.5 ± 16.7	26–58	50	30.2 ± 6.3	24.1–39.0	34.0 ± 16.3	16–48
**Scoliosis**	2	24.0 ± 8.4	18–30	50	24.1 ± 5.5	20.0–28.0	n/a	n/a
**Total**	26	54.23 ± 15.0	18–74	58	26.7 ± 5.3	16.4–39.0	42.9 ± 14.0	16–78

Values are years for age, BMI in kg/m^2^, ODI in percentage. Gender values represent the percentage of male participants in each group (female = 100 − male). N = number of subjects; BMI = body mass index; ODI = Oswestry Disability Index; n/a = not applicable. * ODI was not available for all participants. ODI data were available for 13 patients with lumbar spinal stenosis, 3 with spondylolisthesis, and 3 with lumbar disc herniation; no ODI data were available for the scoliosis subgroup.

**Table 2:** TLF thickness on MRI by pathology.

	TLF Thickness on Axial Plane			TLF Thickness on Sagittal Plane		
Diagnosis	N	Mean ± SD	Range	N	Mean ± SD	Range
**Spinal stenosis**	11	1.01 ± 0.22	0.75–1.35	11	1.27 ± 0.56	0.47–2.33
**Spondylolisthesis**	2	1.11 ± 0.40	0.83–1.40	2	0.96 ± 0.03	0.94–0.98
**Lumbar disc herniation**	5	0.53 ± 0.29	0.16–0.79	5	1.01 ± 0.32	0.63–1.44
**Scoliosis**	1	0.81	n/a	0	n/a	n/a
**Total**	**19**	**0.89 ± 0.33**	**0.16–1.40**	**18**	**1.16 ± 0.48**	**0.47–2.33**

Values are mean ± SD (range), in millimeters. Thickness was measured on axial and sagittal planes. N = number of subjects; SD = standard deviation; n/a = not applicable.

**Table 3:** TLF thickness on US by pathology.

	TLF Thickness on Short Axis (mm)			TLF Thickness on Long Axis (mm)		
Diagnosis	N	Mean ± SD	Range	N	Mean ± SD	Range
**Spinal stenosis**	13	2.83 ± 1.63	1.01–5.91	9	2.65 ± 1.31	1.12–4.49
**Spondylolisthesis**	3	1.35 ± 0.33	1.01–1.67	3	1.98 ± 0.88	1.12–2.87
**Lumbar disc herniation**	2	2.25 ± 0.18	2.13–2.38	2	2.61 ± 1.34	1.66–3.56
**Scoliosis**	1	2.72	n/a	1	2.30	n/a
**Total**	**19**	**2.53 ± 1.44**	**1.01–5.91**	**15**	**2.49 ± 1.14**	**1.12–4.49**

Values are mean ± SD (range), in millimeters. Thickness was measured in short-axis and long-axis views. N = number of subjects per diagnosis; SD = standard deviation; n/a = not applicable.

## Data Availability

The data presented in this study are available on request from the corresponding author due to privacy and ethical reasons.

## Supplementary Materials

The following supporting information can be downloaded at https://www.mdpi.com/article/10.3390/medicina62061045/s1, Table S1: MRI sagittal vs. long-axis US; Table S2: MRI axial vs. short-axis US.
