# Supplementary material for: MRI and Ultrasound of the Thoracolumbar Fascia in the Setting of Degenerative Spinal Diseases
Source: Medicina (Kaunas). 2026 May 28;62(6):1045. doi: 10.3390/medicina62061045 (PMC13303977; doi:10.3390/medicina62061045)
Supplement: Supplementary file 1 [file medicina-62-01045-s001.zip › Supplementary Table S1.pdf]

Supplementary Table S1: MRI sagittal vs long-axis US

	Sample	MRI sagittal	Long-axis ultrasound
1	9 TLF	2.33	3.93
2	18 TLF	0.89	1.86
3	22 TLF	0.46	1.97
4	26 TLF	0.77	2.49
5	41 TLF	1.67	2.11
6	43 TLF	0.58	1.11
7	48 TLF	1.68	4.48
8	33 TLF	0.93	2.87
9	38 TLF	1.16	3.55
10	49 TLF	0.76	1.66
11	36 TLF	1.48	3.72
12	3 TLF	1.12	5.53
