# Supplementary material for: MRI and Ultrasound of the Thoracolumbar Fascia in the Setting of Degenerative Spinal Diseases
Source: Medicina (Kaunas). 2026 May 28;62(6):1045. doi: 10.3390/medicina62061045 (PMC13303977; doi:10.3390/medicina62061045)
Supplement: Supplementary file 1 [file medicina-62-01045-s001.zip › Supplementary Table S2.pdf]

Supplementary Table S2: MRI axial vs short-axis US

| 1 | Sample | MRI axial | Short-axis ultrasound |
|---|---|---|---|
| 2 | 9 TLF | 0.86 | 3.39 |
| 3 | 13 TLF | 1.23 | 4.92 |
| 4 | 18 TLF | 0.98 | 1.72 |
| 5 | 22 TLF | 0.75 | 2.48 |
| 6 | 26 TLF | 0.75 | 1.17 |
| 7 | 37 TLF | 1.33 | 3.27 |
| 8 | 41 TLF | 0.86 | 1.17 |
| 9 | 43 TLF | 0.83 | 1.01 |
| 10 | 48 TLF | 1.14 | 2.24 |
| 11 | 33 TLF | 1.39 | 1.36 |
| 12 | 38 TLF | 0.79 | 2.38 |
| 13 | 49 TLF | 0.15 | 2.12 |
| 14 | 36 TLF | 0.76 | 1.33 |
| 15 | 3 TLF | 1.08 | 2.33 |
